# Insights Into Spatial Synchrony Enabled by Long‐Term Data

**DOI:** 10.1111/ele.70112

**Published:** 2025-04-23

**Authors:** Daniel C. Reuman, Jonathan A. Walter, Lawrence W. Sheppard, Vadim A. Karatayev, Ethan S. Kadiyala, Amanda C. Lohmann, Thomas L. Anderson, Nat J. Coombs, Kyle J. Haynes, Lauren M. Hallett, Max C. N. Castorani

**Affiliations:** ^1^ Department of Ecology & Evolutionary Biology and Center for Ecological Research University of Kansas Lawrence Kansas USA; ^2^ Center for Watershed Sciences University of California, Davis Davis California USA; ^3^ Department of Environmental Sciences University of Virginia Charlottesville Virginia USA; ^4^ Marine Biological Association of the United Kingdom Plymouth UK; ^5^ Department of Biology University of Maryland College Park Maryland USA; ^6^ Department of Biological Sciences Southern Illinois University Edwardsville Edwardsville Illinois USA; ^7^ Department of Biology University of Oregon Eugene Oregon USA

**Keywords:** changes in synchrony, climate change, long time series, spatial synchrony, stability, timescale‐specific, wavelet

## Abstract

Spatial synchrony, the tendency for temporal fluctuations in an ecological variable to be positively associated in different locations, is a widespread and important phenomenon in ecology. Understanding of the nature and mechanisms of synchrony, and how synchrony is changing, has developed rapidly over the past 2 decades. Many recent developments have taken place through the study of long‐term data sets. Here, we review and synthesise some important recent advances in spatial synchrony, with a focus on how long‐term data have facilitated new understanding. Longer time series do not just facilitate better testing of existing ideas or more precise statistical results; more importantly, they also frequently make possible the expansion of conceptual paradigms. We discuss several such advances in our understanding of synchrony, how long‐term data led to these advances, and how future studies can continue to improve the state of knowledge.

## Graphical abstract

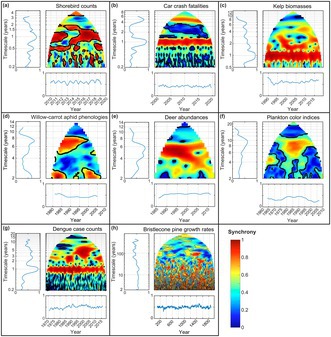

## 1 Introduction

Spatial synchrony is the tendency for temporal fluctuations in an ecological variable—often population abundance—to be positively associated across distinct locations, that is, values in distinct locations tend to rise and fall together. This phenomenon is very common, and conceptually important. The commonness of spatial synchrony is underscored by the wide range of taxa (Liebhold et al. 2004) and physical (Koenig 2002; Magnuson et al. 2005) and biogeochemical (Abbott et al. 2018; Doyle et al. 2019; Magnuson et al. 1990; Seybold et al. 2022) variables in which it has been observed, over distances from centimetres to thousands of kilometres (Koenig 2002; Liebhold et al. 2004). The importance of synchrony (Hansen et al. 2020; Reuman et al. 2023; Schindler et al. 2015; Seybold et al. 2022; Walter et al. 2017; Wang and Loreau 2014) stems partly from its implications for stability. Spatial synchrony enhances the temporal variance—a common measure of instability—of spatially aggregated quantities (the total or average across space) because the synchronous components of local variation reinforce each other when aggregated, leading to large fluctuations (Anderson et al. 2021). For example, total crop yield across an area is more variable if the yields of individual farms fluctuate synchronously. Relatedly, synchrony of populations is thought to heighten extinction risk by reducing the potential for dispersal to rescue populations from local extinction (Heino et al. 1997). Ecologists are also interested in other kinds of synchrony, notably phenological synchrony and community synchrony. Due to similarities of concepts and methods, some points made here also apply to those phenomena, but we focus on spatial synchrony, henceforth ‘synchrony’.

Many conceptual developments over the past decades in our understanding of synchrony came about through the study of long‐term data sets, where ‘long‐term’ is here interpreted as ≥20 years of study (see Section 6 for other interpretations). The purpose of this paper is to synthesise these developments, with a special focus on how long‐term data sets facilitate new understanding. Ecologists are familiar with the idea that higher quality and more extensive data sets provide better tests of existing concepts and more accurate estimates of important quantities. Our thesis goes beyond those expectations. We explore how longer time series have also facilitated new concepts and the expansion of paradigms for understanding synchrony.

Some reflections of Doak et al. (2008) help indicate why long‐term data sets may be important for facilitating discoveries in ecology. To paraphrase, ecosystem dynamics may often be driven by the simultaneous influences of a large number of mechanisms, perhaps a small percentage of which are even known to science. Therefore, any ecosystem, if studied in enough detail (e.g., using long‐term data), may reveal dynamical mechanisms that were previously unknown to science, but may then be found to be generally important, leading to paradigm shifts. Key here is the idea that a detailed study of an ecosystem may often reveal not only mechanisms previously unknown to be important for that system, but also previously unknown to operate in any ecosystem. We revisit these ideas in Section 6.

**FIGURE 1:**
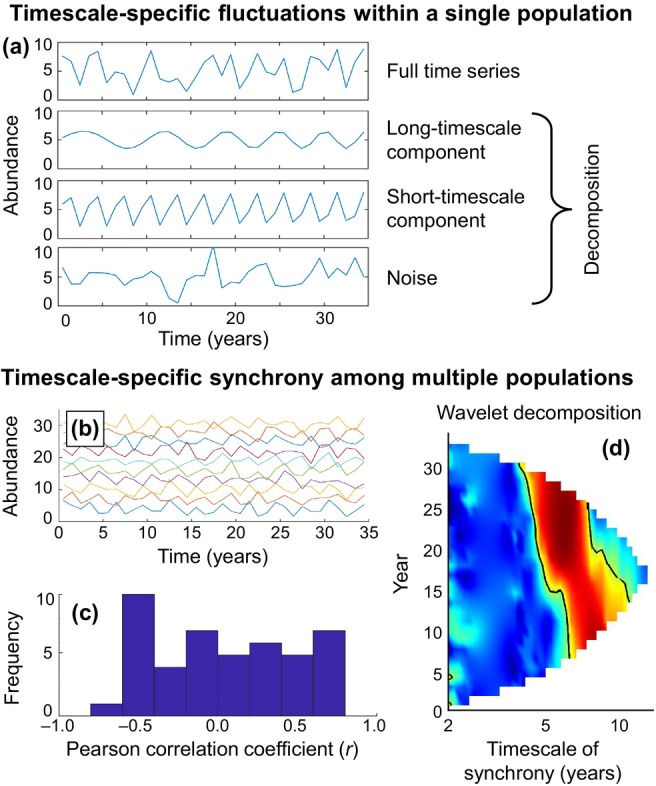
Demonstration of how timescale structure in synchrony can occur and one tool that can help detect it. Panel (a) shows how a single time series can be a superposition of multiple timescale components. This simulated population exhibits both long‐ and short‐timescale fluctuations, plus noncyclical white noise; it is constructed as the mean of these three components. In panel (a), note that the long‐timescale fluctuation (mean period = 7 years) cycles more rapidly over time. We generated 10 time series (panel b) synchronised by this component, but with independently phase‐shifted short‐timescale fluctuations (period = 3 years), and independent noise. Synchrony patterns are not easily visually identified here (b), nor are they detected using conventional pairwise correlations (c); but synchrony is revealed, for instance, by a ‘wavelet phasor mean field’ technique (d) which is among a suite of wavelet tools (e.g., Anderson et al. 2021; Cazelles et al. 2005; Cazelles and Stone 2003; Keitt 2008; Reuman et al. 2021; Sheppard et al. 2016, 2019; Vasseur et al. 2014) now used to study synchrony. The wavelet phasor mean field combines wavelet transforms of multiple time series to reveal aspects of the timescale‐specific structure of synchrony, as well as changes in that structure through time (in this case due to the changing period of the long‐timescale component of panel a). Colours in panel (d) represent intensity of phase synchrony, scaled between 0 and 1, with the black contour line representing significance of phase synchrony (95%). The wavelet phasor mean field is $\frac{1}{N}\sum_{n=1}^{N}p_{n,\sigma }\left(t\right)$ for the ‘phasors’ $p_{n,\sigma }\left(t\right)=w_{n,\sigma }\left(t\right)/|w_{n,\sigma }\left(t\right)|$, where $w_{n,\sigma }\left(t\right)$ is the wavelet transform of the nth available time series evaluated at timescale σ and time t. Significance, here, is tested by comparison to a null hypothesis of random independent phasors (Anderson et al. 2019).

We review and synthesise four trends in the study of synchrony and reflect on what these developments tell us about the value of long‐term data. Our intended audience includes ecologists interested in synchrony or long‐term data sets; no deep knowledge of synchrony is assumed. The first area we review (Section 2) involves the gradual realisation that synchrony generally has a pronounced ‘timescale structure’. It has long been known that population dynamics can be viewed as the superposition of fluctuations on timescales ranging from days to decades or longer (Figure 1a; Inchausti and Halley 2001; Pimm and Redfearn 1988; Sugihara 1995). More recent studies have observed that the dynamics of two or more populations can be synchronised on some timescales while being less synchronised or unrelated on other timescales (Anderson et al. 2019; Broutin et al. 2005; Grenfell et al. 2001; Keitt 2008; Sheppard et al. 2016; Valpine et al. 2010; Vasseur et al. 2014; Vasseur and Gaedke 2007; Viboud et al. 2006). This has become a paradigm for synchrony studies; it now seems difficult to imagine a complete understanding of synchrony without considering timescale structure. Some of the other recent developments we discuss would also have been difficult without a timescale‐conscious approach.

Second, inferences of the likely causes of synchrony have become better statistically supported and widely performed over the last 2 decades (Section 3). It has long been known theoretically that environmental drivers—often climate variables—can induce spatial synchrony of populations through a process known as the *Moran effect* (Moran 1953). Likewise, the theoretical importance of dispersal between populations for causing synchrony was known for decades (Kendall et al. 2000; Molofsky 1994; Ranta et al. 1998). A third potential cause involves a mobile or synchronous predator (de Roos et al. 1998; Ims and Steen 1990), the predator inducing synchrony in a focal species through interactions with it. Historically, it was considered difficult to determine from observations which combination of causes operated in any given situation (Bjørnstad et al. 1999; Liebhold et al. 2004). This was partly because, using historically common approaches, multiple mechanisms could produce similar patterns (Abbott 2007). But multiple new approaches are now increasingly used, alongside other arguments, to support determination of which causes of synchrony dominate in a given system, and to identify environmental drivers of Moran effects.

Third (Section 4), changes through time in the strength, timescale structure, geography or other features of synchrony have emerged as an important topic (Grenfell et al. 2001; Hansen et al. 2020; Kahilainen et al. 2018; Ojanen et al. 2013; Post and Forchhammer 2004; Rohani et al. 1999; Sheppard et al. 2016; Tack et al. 2015; Walter et al. 2017). Historic practices typically relied on correlation‐based measures of synchrony that provide average synchrony estimates, limiting detection of temporal changes in synchrony. Early work indicated the potential importance of changes through time (Kaitala et al. 2001; Koenig 2001; Ranta et al. 1998; Steen et al. 1990). Accelerating work over the past decade has shown that changes in synchrony are common and are probably another widespread impact of climate change (Black et al. 2018; Di Cecco and Gouhier 2018; Kahilainen et al. 2018; Koenig and Liebhold 2016; Sheppard et al. 2016; Tack et al. 2015; reviewed by Hansen et al. 2020).

Fourth, long‐term data have facilitated new insights into mechanisms of synchrony and related processes (Section 5). To illustrate, we provide three examples well known to us. Though the Moran effect (Moran 1953) has long been known, recent studies have revealed how environmental drivers of spatial synchrony can interact, thereby either enhancing or diminishing synchrony compared to the synchrony that would have occurred without interactions (Castorani et al. 2022; Reuman et al. 2023; Sheppard et al. 2019). Many researchers have noted the expectation that multiple Moran drivers may act on a set of populations, but only recently has a formal theory of interactions been developed. In a second example, timescale‐specific synchrony was recently found to beget cyclic patterns in regional population abundances (Anderson et al. 2021). This mechanism is superficially opposite to the long‐known link between cycles and synchrony by which locally cyclic dynamics beget synchrony because cyclic systems can often be easily synchronised. Lastly, long‐term data have shown how synchrony can be ‘tail‐dependent’, i.e., stronger when populations are jointly abundant versus jointly rare, or vice‐versa (Ghosh et al. 2021; Ghosh, Sheppard, Holder, et al. 2020); and how tail‐dependent synchrony can arise (Walter et al. 2022). While these are far from the only discoveries in recent years about synchrony mechanisms, they will serve as illustrations of the field's progress, enabled by long‐term data.

In all cases, ideas are advanced principally through consideration of case studies with which the authors are most familiar, though we also attempt to briefly review relevant literature. In each section, we discuss how ideas were not merely better tested or refined, but had their conceptual origin through the examination of long‐term data.

## 2 Timescale Structure of Synchrony

### 2.1 Overview of Timescale Structure

Recent progress in synchrony has been enabled by systematic consideration of the different timescales of variability present in ecological data, each of which may have its own associated mechanisms or drivers, and each of which may thus exhibit a different pattern of synchrony. The *characteristic timescale* of a cyclic fluctuation (e.g., a sinusoid) is the period of the oscillation. Because any time series can be viewed as the superposition of fluctuations operating on a range of timescales (Figure 1a), variability in a time series can be represented by a *power spectrum*, which indicates the magnitude of variation present at each timescale. A strong regular fluctuation, such as annual variability, will be associated with a significant peak in the power spectrum at the characteristic timescale. The power spectrum of a time series sampled at times $0,\Delta ,2\Delta ,\ldots ,\left(L-1\right)\Delta$ provides information about fluctuations at timescales ranging from 2Δ up LΔ, and other spectral methods are similarly limited (see Section 2.4 for additional details on the 2Δ timescale bound). Many ecological processes are positively temporally autocorrelated and dominated by fluctuations at longer timescales (Pimm and Redfearn 1988; Rudnick and Davis 2003; Sugihara 1995) and so are described as having a ‘red spectrum’ (Rudnick and Davis 2003), because red light has longer wavelengths than other colours of visible light.

Synchrony between two or more time series can analogously be decomposed by timescale (Keitt 2008; Vasseur and Gaedke 2007), and synchrony can differ in strength across timescales (Figure 1). By decomposing synchrony by timescale, we can identify synchrony that may have been undetectable to traditional, correlation‐based measures of synchrony because it was obscured by asynchronous dynamics at other timescales. For instance, populations of many species are synchronised over broad spatial extents at long timescales (e.g., decades) by low‐frequency climate oscillations such as the El Niño Southern–Oscillation (ENSO; Anderson et al. 2021; Cazelles et al. 2005; Lara et al. 2019) or the North Atlantic Oscillation (NAO; Grøtan et al. 2005; Post and Forchhammer 2002; Sheppard et al. 2016), even though such long‐timescale synchrony can be obscured by idiosyncratic local factors which contribute asynchronous fluctuations on shorter timescales (Figure 1). We henceforth say that synchrony in a set of time series has ‘timescale structure’ if synchrony is stronger on some timescales than others.

### 2.2 Timescale Structure in Synchrony Is Important and Common

Numerous papers have now collectively revealed that meaningful timescale structure in synchrony is common (e.g., Anderson et al. 2019; Bjørnstad et al. 2008; Castorani et al. 2022; Cazelles et al. 2005; Chavez and Cazelles 2019; Cooke and Roland 2023; Emery et al. 2023; Grenfell et al. 2001; Keitt 2008; Reuman et al. 2023; Sheppard et al. 2016, 2019; Valpine et al. 2010; Vasseur et al. 2014; Vasseur and Gaedke 2007; Viboud et al. 2006; Walter et al. 2024, 2021). Spectral methods have been used in ecology for decades (Bartlett 1954; Cazelles et al. 2008; Platt and Denman 1975; Rouyer et al. 2008), including in studies of synchrony (Grenfell et al. 2001; Viboud et al. 2006); and several studies have also prefigured the formal statistical consideration of timescales in synchrony (e.g., Kitzberger et al. 2011; Stenseth et al. 2004). Recent work has also benefited from methods that explicitly study synchrony among many time series and drivers thereof (Chavez and Cazelles 2019; Sheppard et al. 2016, 2019), rather than assessing time series one at a time or in pairs. Collectively, all this work has shown that it is common for synchrony to be stronger on some timescales than on others, with specific patterns differing from system to system in ways that can reveal aspects of population dynamics.

**FIGURE 2:**
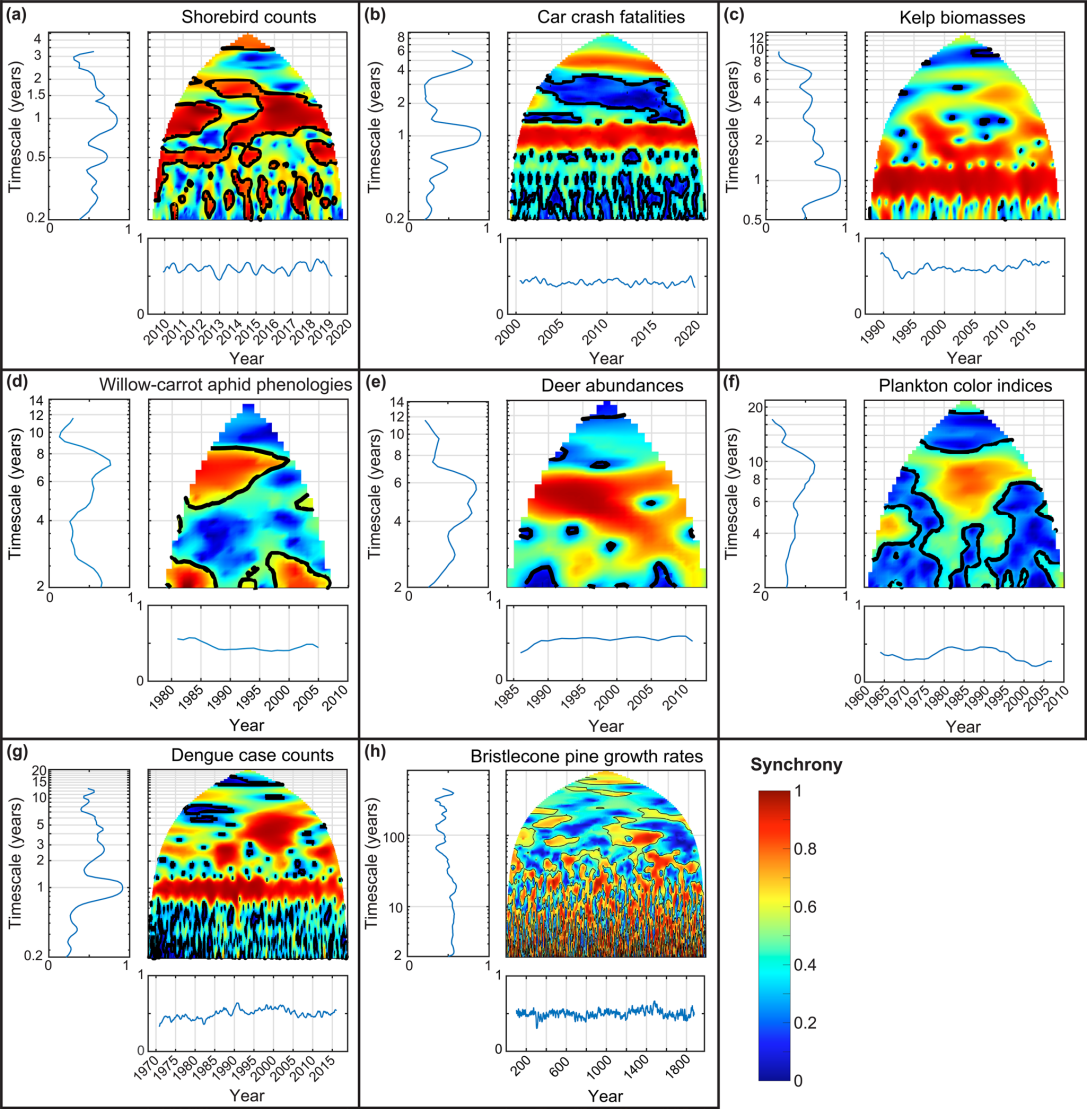
Prominent timescale structure in synchrony has been explored in a diversity of systems using long‐term data. We here provide some examples. Each panel shows a wavelet phasor mean field, with a significance threshold contour (95%). See Figure 1 for a demonstration of the wavelet phasor mean field technique. Side panels are averages of each main panel across times or timescales. Data were: (a) monthly shorebird counts at 11 beach sites in southern California, USA (Walter et al. 2024); (b) monthly car crash deaths in 41 of the contiguous 48 United States, from the Multiple Cause of Death database, 1999–2020, via the Centers for Disease Control and Prevention Wonder database (Section S1); (c) monthly time series of kelp biomass in 242 locations (500 m stretches of coastline) along the coast of central California, USA (Castorani et al. 2022); (d) annual first‐flight dates of the willow‐carrot aphid ( *Cavariella aegopodii* ) observed at 11 sites across the UK, from the Rothamsted Insect Survey (Sheppard et al. 2016); (e) annual deer abundance time series in 71 counties in Wisconsin, USA, from the Wisconsin Department of Natural Resources (Anderson et al. 2021); (f) annual time series of phytoplankton abundance as measured by a colour index, from 26 areas, each 2° × 2°, in UK seas, from the Continuous Plankton Recorder Survey (Sheppard et al. 2019); (g) monthly dengue case counts for 72 of the provinces of Thailand, provided by the Thai Ministry of Public Health in their Annual Epidemiological Surveillance Reports (García‐Carreras et al. 2022); and (h) annual ring width index (i.e., growth) time series from 9 bristlecone pine groves in California, Nevada, and Utah, USA, for 1980 years, from the International Tree‐Ring Data Bank (Section S1). Each panel, except for b and h, is a place holder for detailed statistical analyses, reported in the references and differing in nature from system to system, supporting the claim that timescale structure of synchrony in these systems was meaningful and important. Panels (b) and (h) are new, but show similar patterns. Black contours separate plots into a region for which synchrony was significant (containing the reddest colours) and a region where it was not significant (coolest colours) according to the wavelet phasor mean field technique. In some cases, contours include all but the coolest colours. See Section S2 for additional details.

The examples in Figure 2 are taken from publications examining synchrony of time series from marine, freshwater and terrestrial systems and depict spatial synchrony of population counts, densities or biomass estimates; phenological variation (dates); growth rates; and epidemiological case counts. Numerous patterns are apparent, including temporally consistent synchrony occurring principally on specific timescale bands (Figure 2b, 1 year and 4–6 year bands; e, 3–7 year bands; c and g, 1 year bands) and long‐lasting but ultimately transient patterns of synchrony on timescales from 5 years (g) to 5–10 years (f) to 100 years (h). Synchrony on annual timescales is common because of seasonality, but temporally consistent synchrony on multi‐annual timescales is also common. Synchrony often occurs consistently through time and at statistically significant levels (according to the wavelet phasor mean field methods of Figure 1, also employed in Figure 2) in two timescale bands while dropping to nonsignificant levels in between (e.g., Figure 2b). It is important to note that wavelet mean field plots of Figure 2 do not stand alone to demonstrate statistically significant timescale structure in synchrony in the corresponding systems. Panels should be considered illustrations of earlier results, most backed by detailed investigations described in the corresponding references. The nature of some of these detailed investigations is summarised in Section S3.

We note that several methods have been proposed for quantifying timescale‐specific synchrony from population time series, including the wavelet phasor mean field used here, and others (Brillinger 2001; Chavez and Cazelles 2019; Keitt 2008; Rouyer et al. 2008). Methods are evolving, and addressing nonstationarity in data while codifying an appropriate null hypothesis against which to compare the apparent structure of synchrony in the data is part of the challenge (Chavez and Cazelles 2019). Nevertheless, the conclusion appears to be robust that synchrony often occurs on some timescales of analysis while being absent or weaker on others. For instance, Chavez and Cazelles (2019) compared several methods, considering simulated and empirical data sets. Though the precise portions of time‐timescale space which showed significant synchrony differed somewhat among their methods, all methods consistently showed that significant synchrony occurred only on some timescales. The wavelet phasor mean field used for illustration throughout this paper quantifies average synchrony across all the time series analysed. High values mean that many, but not necessarily all, of the time series exhibit synchronous dynamics.

### 2.3 Benefits of Long‐Term Data for Quantifying Timescale‐Specific Synchrony

**FIGURE 3:**
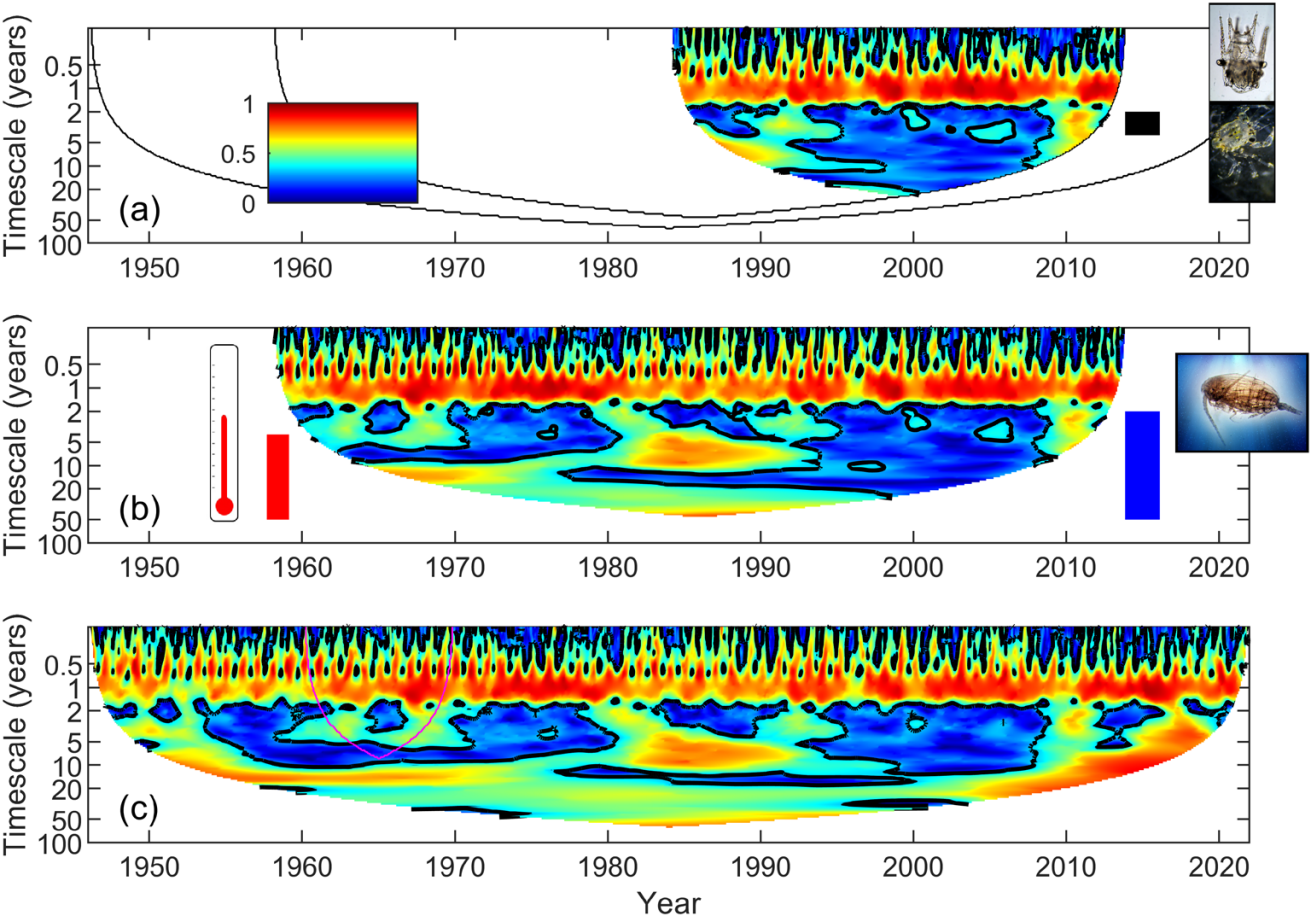
Synchrony in the phytoplankton colour index (PCI), a colour‐based index of bulk phytoplankton density, in seas around the UK, using (a) data from 1984 to 2013, (b) data from 1958 to 2013, (c) data from 1946 to 2021. Panels show synchrony via the wavelet phasor mean field, with a black contour indicating statistical significance at the 95% level or above (Figure 1), for time series of PCI in 26 locations around the UK. Sheppard et al. (2019) used data from 1958 to 2013 to establish drivers of synchrony (panel b); the shorter and longer time series plots are presented for comparison. Black outlines on panel (a) correspond to the boundaries of the plots on (b) and (c), to facilitate comparisons. The magenta line on panel (c) corresponds to the plot boundaries that would have occurred if data spanned only 1960–1970. Echinoderm larvae and decapod larvae abundances predicted variation of PCI in the 2−4 year timescale band (this timescale range is spanned by the black bar in panel a), sea surface temperature predicted variation in the 4+ year timescale band (this range is spanned by the red bar in panel b), and *Calanus finmarchicus* abundance predicted variation across both bands (this range is spanned by the blue bar in panel b). These results were based on the data of Johns (2023).

Figure 2 reveals benefits of long time series for studies of synchrony, starting with phytoplankton density data, analysed in Figure 2f and examined in greater detail now. The Continuous Plankton Recorder (CPR) survey (Batten et al. 2003; Colebrook and Robinson 1965; Raitsos et al. 2014) has operated since prior to World War II. Data considered here come from monthly measurements of a phytoplankton colour index (PCI) in 26 areas in the seas around the UK from 1946 to 2021. PCI corresponds with estimates of bulk phytoplankton density (Joint and Pomroy 1993; McClain 2009; Raitsos et al. 2014, 2013). For Figure 3, CPR time series were truncated to various lengths for illustration. Panel a truncates data to half the length which was available to Sheppard et al. (2019), panel b truncates data to what was available at the time of the analysis, and panel c incorporates all data available now. The outer black lines on panel a correspond to the boundaries of the wavelet phasor mean fields of the other panels. Comparing the panels of Figure 3, and the black lines on panel a, shows that increasing the length of time series increases not only the time over which we can track synchrony for a given timescale, but also the range of timescales we can examine, and substantially so (note the log scale on the timescale axes). For instance, the maximum timescale which can be examined on panel a is about 24 yrs.; maximum timescales on panels b and c are about 46 and 64 yrs. Because time series length increases both the times and timescales which can be examined, it may be argued that the value of time series data grows not linearly, but closer to the square of time series length.

To illustrate, longer PCI time series reveal a long‐timescale synchronous event spanning from the 1950s to the early 1970s (Figure 3c, lower left). This event occurred on timescales greater than 10 years, so could not have been revealed with short sampling efforts, even if those efforts overlapped the feature in their timing. A magenta outline on Figure 3c corresponds to the boundary of the wavelet phasor mean field that would occur if data were available from 1960 to 1970. Though that sampling period occurred during the event, data of that duration would not reveal the event because the event involved timescales that could not be assessed using time series of the given length. The longest data (Figure 3c) also suggest that annual‐timescale synchrony has intensified since the 1940s.

In addition to the lessons above, strong evidence also emerges from the history of timescale‐conscious approaches to synchrony, showing that long time series have been crucially important for expanding conceptual paradigms. Early applications of wavelets in ecology used very long time series. Three examples involved weekly measles (Grenfell et al. 2001) and influenza (Viboud et al. 2006) data sets spanning 1944–1994 and 1972–2002, respectively; and monthly dengue reports spanning 1983–1997 (Cazelles et al. 2005). Later applications to nonepidemiological data also relied on long time series, e.g., the data of Keitt (2008) were 17 years long, sampled every 2–6 weeks; and the data of Sheppard et al. (2016) spanned 35 years. Although these time series were long for ecology, wavelet tools were more frequently applied in fields such as biophysics, where researchers consider wavelets to be tools most suitable for even longer time series (Bandrivskyy et al. 2004; Stefanovska and Hožič 2000). Wavelet tools therefore may not have been applied to study ecological synchrony were it not for the availability of long time series; and researchers may then have been delayed in their realisation of the importance of timescale structure in synchrony. Whereas historic use of lagged correlograms to study synchrony (Bjørnstad et al. 2002, 1999; Buonaccorsi et al. 2001) indicates that researchers were aware of the potential for timescale structure in synchrony, wavelet methods reveal such patterns more directly than correlograms (e.g., Keitt 2008; Sheppard et al. 2016; Figures 1, 2, 3 of this paper). It seems to us that such clear depictions substantially accelerated the paradigm shift whereby now timescale structure in synchrony is widely appreciated.

Finally, we note that the bristlecone pine example underlying Figure 2h shows that, for that system, there is no evidence of a timescale above which additional synchrony ceases to be revealed by longer data. Many other systems also show prominent synchronous features on the longest timescales that can be examined.

### 2.4 The Importance of Sampling Interval

Just as time series length limits the longest timescales on which synchrony (or any other dynamical phenomenon) can be studied, the time interval between samples also limits the shortest timescales. Although we here focus on time series length, this mirror‐image limitation should not be forgotten. The well‐known ‘Nyquist frequency’, equal to half the sampling frequency, is the highest frequency for which Fourier or wavelet methods can provide information. Hence, the shortest timescale which can be assessed is double the sampling interval. Annual sampling cannot reveal information about periodicities shorter than 2 years which may be present in the focal system. As another example, the sardine and anchovy time series of Baumgartner et al. (1992), reconstructed from sediment cores and remarkable for their length of well over 1000 years, do not facilitate study of sardine or anchovy population periodicities less than 20 years because the sampling interval was 10 years, due to resolution limitations of the sediment core methods used. Long‐term studies aimed at understanding synchrony should carefully consider the frequency as well as the duration of sampling.

## 3 Inferring the Causes of Synchrony

### 3.1 Historical Versus Modern Approaches to Inference

Classically, it was considered difficult to infer the causes of synchrony in nature (Abbott 2007; Bjørnstad et al. 1999; Liebhold et al. 2004). However, statistical developments have led to approaches capable of facilitating such inferences (e.g., Defriez and Reuman 2017; Gouveia et al. 2016; Haynes et al. 2013; Lara et al. 2019; Nicolau et al. 2022; Raimondo et al. 2004; Sheppard et al. 2016, 2019; Walter et al. 2017; Wanner et al. 2024). Researchers have now generally accepted the expanded paradigm that main causes of synchrony should often be discernible.

Historically, to infer the primacy of one cause of synchrony, ecologists often relied on: (1) special cases, where certain causes of synchrony could be ruled out; (2) interspecific comparative approaches; or (3) preliminary inferences based on synchrony‐distance relationships. As an example of 1, synchrony among feral sheep populations on Scottish islands was determined to arise from Moran effects, as dispersal was infeasible, and predators were absent (Grenfell et al. 1998; see also Post and Forchhammer 2002). As an example of 2, Paradis et al. (1999) inferred that dispersal was important for the synchrony of British birds by observing that more broadly dispersing species tended to exhibit stronger synchrony (see also Peltonen et al. 2002; Tedesco and Hugueny 2006). As an example of 3, Økland and Bjørnstad (2003) hypothesised that spatial synchrony in tree windfalls may drive synchrony of the spruce bark beetle (*Ips typographus*), largely because patterns of decline in synchrony with distance were similar for windfall and beetles. But their hypothesis seemed tentative, perhaps appropriately, since it was later demonstrated that distinct synchrony causes can produce similar patterns of decline with distance (Abbott 2007); and declines are also influenced by local dynamics in a confounding manner (Bjørnstad et al. 1999; Ranta et al. 1998). While the approaches described above can sometimes provide powerful evidence, the field lacked generalisable analytical approaches (Bjørnstad et al. 1999; Liebhold et al. 2004), which have since developed, for inferring synchrony causes.

One class of approaches exploits a timescale‐specific framework (Cazelles et al. 2005; Grenfell et al. 2001; Lara et al. 2019; Reuman et al. 2021; Sheppard et al. 2019). In an early example, wavelet analysis was used to identify time lags between peaks of measles incidence in major population centres versus smaller towns. The dependence of these lags on distance indicated that transmission dynamics (dispersal), not Moran effects, primarily drove synchrony in measles outbreaks prior to vaccines (Grenfell et al. 2001). This approach focused on phase lags at the specific timescale of outbreaks. Other early and influential papers adopting a timescale‐specific approach include Cazelles et al. (2005), Cazelles and Stone (2003), Keitt (2008), and Viboud et al. (2006); and Buttay et al. (2022) and Churakov et al. (2019) are recent examples using similar tools.

These works helped inspire the development of a now fairly well developed suite of methods based on a tool called *spatial wavelet coherence*, which tests whether the phase differences between timescale‐specific oscillations in a set of biological time series (e.g., population abundance at multiple locations) and a set of climatic time series (e.g., rainfall across the same locations) are more consistent than would be expected by chance if the two corresponding variables were unrelated (Cazelles et al. 2005; Lara et al. 2019; Sheppard et al. 2016). This technique extends the classical wavelet coherence, used to test for relationships between pairs of time series (Cazelles et al. 2008, 2005), to the context of spatiotemporal variables. Coherence can occur on some timescales while being absent on others if, for instance, population vital rates are driven by climate via a moving average process (see Supplementary figure S5 of Sheppard et al. 2016). Given that it is frequently safe to assume that the biological quantities of interest are not influencing climate, a highly significant spatial wavelet coherence suggests that the climatic variable is driving the oscillations of, and hence transmitting synchrony to, the biological time series (Sheppard et al. 2016). It is also possible that the climatic variable influenced the population variable indirectly, or that it is closely related to another, unmeasured climatic variable that is the underlying cause of population synchrony; though these cases still indicate that a Moran effect occurred. Biological arguments and phase relationships are typically used to further substantiate inferences. Other methods such as convergence cross mapping (Clark et al. 2015; Sugihara et al. 2012) and Fourier Granger causality (Dhamala et al. 2008) may also be very effective here. These approaches have demonstrated that environmental causes of synchrony can differ among timescale bands. The basic coherence tool has been extended with a wavelet linear modelling framework, a wavelet Moran theorem, and a synchrony attribution theorem (Reuman et al. 2021; Sheppard et al. 2019). Together, these tools make it possible to identify multiple simultaneous environmental drivers of synchrony and to quantify the fractions of synchrony attributable to each driver and their interactions (Castorani et al. 2022; Reuman et al. 2021, 2023; Sheppard et al. 2019).

The tools described above have produced insights into timescale‐specific synchrony in wide‐ranging systems (Anderson et al. 2019, 2021; Emery et al. 2023; García‐Carreras et al. 2022; Walter et al. 2024). For instance, nutrient availability, wave disturbance and their interaction explained synchrony in giant kelp ( *Macrocystis pyrifera* ) populations, with 65%–67% of synchrony explained on long interannual timescales (4–10 year) and 29%–57% explained on annual timescales (<2 year), depending on region (Castorani et al. 2022). Extreme winters, temperatures during larval development and crop planting dates explained spatial synchrony in an agricultural pest in Sweden (Emery et al. 2023); these variables explained 91% of long‐timescale (7–11 year) synchrony, but only 30% of short‐timescale (2–4 year) synchrony, suggesting that causes of synchrony differed across timescales.

The wavelet tools described above are far from the only methods developed over the last 2 decades to help infer causes of synchrony. Another class of methods exploits detailed spatial variation in synchrony (Defriez and Reuman 2017; Gouveia et al. 2016; Haynes et al. 2013; Klemona et al. 2006; Walter et al. 2017). These approaches capitalise on spatially rather than temporally extensive data. Timescale methods can also be combined with spatial approaches (e.g., Churakov et al. 2019). While integration of data and models has long been used to study synchrony (e.g., Bjørnstad et al. 2008; Buttay et al. 2022; Cattadori et al. 2005; Engen et al. 2005; Grenfell et al. 1998, 2001; Grøtan et al. 2005; Moran 1953), the growing availability of approaches for fitting complex spatiotemporal models to data has provided additional capacity to infer synchrony drivers (e.g., Bouchard et al. 2022; Nicolau et al. 2022; Phillips et al. 2023). It is useful to have multiple means of inferring drivers because they have complementary strengths and can be used jointly to bolster conclusions.

### 3.2 Benefits of Long‐Term Data for Inferences of Causes of Synchrony

The results above imply that longer time series can not only produce incremental improvements in inference; they can also make possible inferences of causes of synchrony which cannot be detected with short time series. Unsurprisingly, longer time series render more robust inferences about the causes of short‐timescale synchrony. But the results summarised above also demonstrated that: (1) drivers of synchrony can differ by timescale, so that drivers of long‐timescale synchrony differ from those of short‐timescale synchrony (previous section); and (2) long‐timescale synchrony can only be explored with long time series (Figure 3). Thus, long time series are necessary to discover causes of synchrony operating on long timescales. Sheppard et al. (2019) inferred that sea surface temperature (likely operating indirectly), a copepod consumer *Calanus finmarchicus* , and their interactions, were drivers of long‐timescale (>4 years) synchrony in phytoplankton in seas around the UK, though these factors did not both drive short‐timescale synchrony (Figure 3a,b). The drivers uncovered in that paper are depicted visually in Figure 3 alongside coloured bars demarcating the range of timescales at which they are significant drivers of synchrony. This and other new approaches to inference illustrate the expanding paradigm whereby inferences of causes of synchrony, previously believed to be difficult, should now be regarded as possible.

## 4 Changes in Synchrony Through Time

### 4.1 Overview of Changes in Synchrony

Long‐term data have been pivotal in demonstrating ways that synchrony can change over time. Understanding how and why synchrony changes through time is important because synchrony is fundamentally related to spatial stability and regional population persistence (Anderson et al. 2021; Heino et al. 1997). Indeed, increases over time in spatial autocorrelation may provide a generic indicator of impending regime shifts (Kéfi et al. 2014), and in at least one instance changes in synchrony were related to ecosystem regime shifts (Defriez et al. 2016). Temporal changes in synchrony may manifest gradually (Black et al. 2018; Choisy and Rohani 2012; Kahilainen et al. 2018; Koenig and Liebhold 2016; Ojanen et al. 2013; Ranta et al. 1998; Shestakova et al. 2016; Tack et al. 2015), abruptly (Cooke and Roland 2023; Defriez et al. 2016; Krebs et al. 2013), or represent oscillations between modes of synchrony and asynchrony (Allstadt et al. 2015; Brommer et al. 2010; Cazelles et al. 2001; Henttonen et al. 1987; Ranta, Kaitala and Lindstrom 1997; Ranta, Kaitala and Lundberg 1997; Vindstad et al. 2019).

Changes in synchrony have sometimes been attributed to changes in external drivers, typically climate variables (Allstadt et al. 2015; Defriez et al. 2016; Kahilainen et al. 2018; Koenig and Liebhold 2016; Läänelaid et al. 2012; Larsen et al. 2024; Sheppard et al. 2016; Tack et al. 2015). However, changes in biotic factors can also lead to changes in synchrony (e.g., changes in dispersal, Choisy and Rohani 2012); and episodic changes can be an emergent outcome of the spatial structure of populations. For example, differences in density‐dependent population regulation underpin some geographic patterns of synchrony (Liebhold et al. 2006; Walter et al. 2017) and explain interspecific differences in the magnitude of synchrony (Marquez et al. 2023). So changes in population regulation—perhaps due to a new competitor or natural enemy—can also lead to changes in synchrony (Matter and Roland 2010). In two early papers, Henttonen et al. (1987) and Steen et al. (1990) argued that changes in the synchrony of rodent populations may have been due to changes in predation. Snowshoe hares ( *Lepus americanus* ) were broadly synchronous across northwestern North America for at least 2 decades, until the mid‐1990s when populations became asynchronous, possibly due to a travelling wave of hare predators (Krebs et al. 2013). Spatially patchy pest outbreaks may cause the growth of aspen ( *Populus tremuloides* ) to become periodically unsynchronised, overriding the synchronising effect of precipitation (Cooke and Roland 2023). Under appropriate dynamical regimes, synchrony can come and go episodically as an emergent property of the spatial arrangement of a system (Cazelles et al. 2001; Ranta, Kaitala and Lindstrom 1997; Ranta, Kaitala and Lundberg 1997).

**FIGURE 4:**
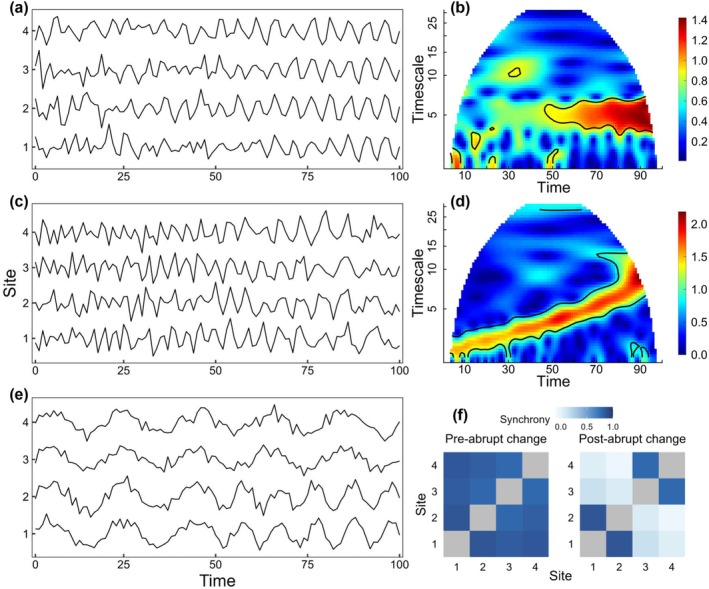
Changes in synchrony of three types, demonstrated using idealised time series based on sinusoidal functions with random noise components. Each time series represents a geographically distinct site that is sampled once per unit time (e.g., annually) for 100‐time steps (e.g., years). Panel (a) shows changes in the strength of synchrony: Four locations that initially exhibit no synchrony but begin to fluctuate in unison at a period of 5 years due to decreasing noise and an increasing sinusoidal component. This is reflected in panel (b) with a wavelet mean field depicting synchrony increasing over time at the 5‐year timescale band. Next, panel (c) shows changes in the timescale structure of synchrony: The dominant timescale of synchrony shifts to longer periods over time, caused by modifying the frequency of the sinusoidal functions. This is reflected in panel (d) with a wavelet mean field. The synchronous signal begins at a period of 2 years and increases to a period of 10 years. Black contours in panels (b) and (d) indicate significant synchrony at a given time and timescale. Lastly, panel (e) depicts changes in the geography of synchrony: Four sites exhibit synchrony until experiencing an abrupt change at sites 1 and 2 halfway through the time series. This is demonstrated in panel (f) with two Pearson correlation matrices, visualising among‐site patterns in synchrony before and after the abrupt change. Correlation matrices are used in f because they demonstrate geographies of synchrony in a manner that mean fields cannot.

Here we focus on three ways synchrony can change through time, underpinned by long‐term empirical evidence: changes in the strength of synchrony (Figure 4a,b), changes in the timescale structure of synchrony (Figure 4c,d) and changes in the geography, or spatial structure, of synchrony (Figure 4e,f).

### 4.2 Changes in the Strength of Synchrony Through Time

Long‐term data instigated many of the major insights into changes in the strength of synchrony over time (Allstadt et al. 2015; Black et al. 2018; Bogdziewicz et al. 2017; Choisy and Rohani 2012; Cooke and Roland 2023; Defriez et al. 2016; Grenfell et al. 2001; Hansen et al. 2020; Henttonen et al. 1987; Johnson and Haynes 2023; Kahilainen et al. 2018; Koenig and Liebhold 2016; Larsen et al. 2024; Ojanen et al. 2013; Post and Forchhammer 2004; Ranta, Kaitala and Lindstrom 1997; Ranta, Kaitala and Lundberg 1997; Shestakova et al. 2016; Steen et al. 1990; Tack et al. 2015). In an early study using several 79‐year time series, Steen et al. (1990) showed that the usually strong synchrony of small rodent dynamics across Norway was temporarily disrupted in the early 1900s (see also Henttonen et al. 1987). The mechanisms behind intermittent synchrony were subsequently explored with a combination of long‐term hare–lynx ( *Lynx canadensis* ) data and theory (e.g., Cazelles et al. 2001; Ranta, Kaitala and Lindstrom 1997; Ranta, Kaitala and Lundberg 1997). Importantly, the empirical discovery of changing or intermittent synchrony in long time series is often what led to subsequent theoretical efforts to explain those patterns.

An early demonstration of directional trends in synchrony compares two records of fur trading and harvest of caribou ( *Rangifer tarandus* ) to show how increased temperatures correlate with increased synchrony of caribou populations (Post and Forchhammer 2004). Other studies of directional change in synchrony have also centred on extraordinarily long direct observations, such as multidecadal insect records (Kahilainen et al. 2018); a very long‐running bird survey (the Christmas Bird Count; Koenig and Liebhold 2016); and growth patterns in tree rings and the calcified structures of bivalves, fish and corals, which can span centuries (Black et al. 2019, 2018; Shestakova et al. 2016).

Across this wide variety of ecosystems exhibiting directional trends through time, synchrony appears most commonly to be increasing, probably as a result of climate change‐related increases in the synchrony of weather patterns (Black et al. 2018; Hansen et al. 2020; Johnson and Haynes 2023; Kahilainen et al. 2018; Koenig and Liebhold 2016; Shestakova et al. 2016). For instance, increasing synchrony of tree growth matches increasing climatic synchrony across Eurasia and the west coast of North America (Black et al. 2018; Shestakova et al. 2016). Increasing metapopulation synchrony of Glanville fritillary butterflies (*Melitaea cinxia*) over the past 2 decades matches increasing synchrony in weather conditions (Kahilainen et al. 2018; Ojanen et al. 2013; Tack et al. 2015), and similar changes threaten to increase the frequency and severity of synchronised forest insect outbreaks (Johnson and Haynes 2023). Lastly, increasing synchrony of temperatures is correlated with an increase in continental‐scale synchrony of North American bird populations (Koenig and Liebhold 2016).

### 4.3 Changes in the Timescale Structure of Synchrony Through Time

The timescale structure of synchrony can also change through time (Anderson et al. 2021; Cazelles et al. 2005; Choisy and Rohani 2012; Defriez et al. 2016; García‐Carreras et al. 2022; Grenfell et al. 2001; Sheppard et al. 2016, 2019; Viboud et al. 2006), meaning that there is a shift in the timescales at which synchrony occurs. Such shifts may be gradual (e.g., Figure 4b,d) or abrupt (e.g., Figure 2d,f,g) relative to time series length and system characteristics such as generation time. As an example of sudden changes in the timescale structure of synchrony, Sheppard et al. (2016) showed that the dominant timescales of synchrony in aphid phenology (day of first flight) in the UK shifted from long timescales (4–20 year) to short timescales (2–4 year) in the early 1990s in response to a change in the dominant periodicities of winter temperature synchrony, which in turn was linked to a shift in the NAO. Thai dengue case counts showed an increase in synchrony at ≈ 2–7 year timescales in the 1990s, and an apparent decline in synchrony at 1‐year timescales at about the same time (Figure 2g; García‐Carreras et al. 2022). An earlier study of the same phenomenon (Cazelles et al. 2005) attributed temporal variability in synchrony to a nonstationary relationship between El Niño and dengue incidence. Additionally, synchrony of marine phytoplankton indices increased during 1975–1995, primarily at 5–10 year timescales (Figure 2f; Defriez et al. 2016).

Studies focusing on changes over time in the timescale structure of synchrony are still few, owing partly to the relative rarity of suitably long time series. However, it seems reasonable to hypothesise that changes in the timescale structure of synchrony have occurred or will occur in many systems because several major climate modes which broadly impact ecosystems (e.g., the NAO and ENSO) and display timescale structure have changed or are forecast to change (e.g., Cai et al. 2014).

### 4.4 Changes in the Geography of Synchrony Through Time

The strength of synchrony can vary geographically in complex ways, for example, certain areas can be more synchronous than others due to a variety of environmental and population‐dynamic factors (Choisy and Rohani 2012; Dallas et al. 2020; Defriez and Reuman 2017; Gouveia et al. 2016; Haynes et al. 2013; Viboud et al. 2006; Walter et al. 2017). Studies employing long‐term data have revealed that such geographic patterns of synchrony can change over time. Some studies have examined how relationships between the strength of population synchrony and the distance between locations have changed over time and whether these changes are the result of changes in the spatial scale of synchrony in abiotic conditions (weather conditions or sea surface temperatures; Bouchard et al. 2022; Defriez et al. 2016; Kahilainen et al. 2018; Koenig and Liebhold 2016; Liebhold et al. 2022). As mentioned above, Glanville fritillary butterfly populations have become more synchronous in parallel with increasing synchrony in weather conditions; but synchrony also increased more between nearby populations than between more distant populations (Kahilainen et al. 2018), so geographies of synchrony also changed. In contrast, for two marine plankton species, synchrony increased more over long distances than short distances during a climate‐driven regime shift (Defriez et al. 2016). Increases in the spatial scale of synchrony, as have been predicted by some climate models (Di Cecco and Gouhier 2018), would have important implications for species conservation by reducing spatial stability over broader areas, increasing extinction risk.

**FIGURE 5:**
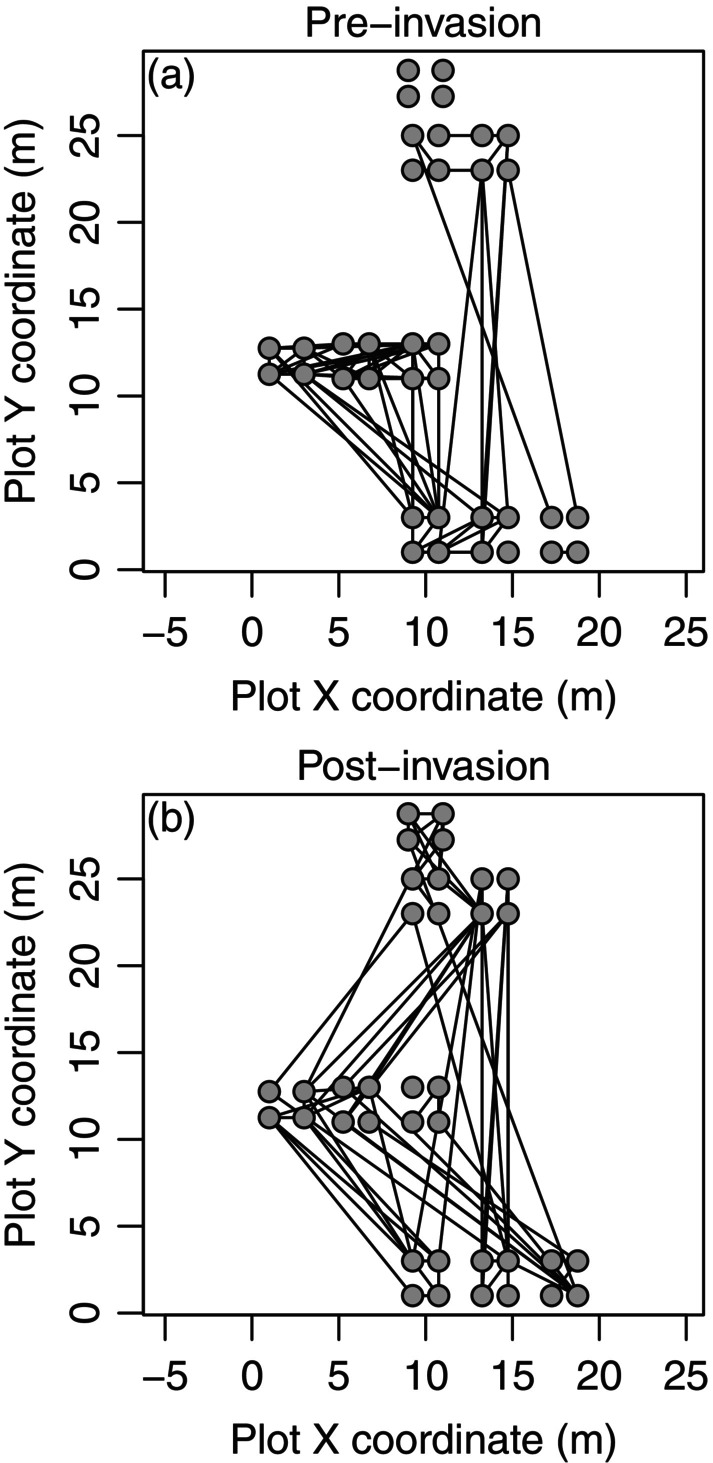
Maps showing changing geographies of synchrony in the forb *Plantago erecta* in Jasper Ridge Biological Preserve following invasion by the non‐native grass *Bromus hordeaceus* . Synchrony networks (Walter et al. 2017) represent plot locations as network nodes and pairwise synchrony between plots as links (edges). Synchrony was measured using Pearson correlation during two equal‐length periods prior to (1983–2000; panel a) and following (2002–2019; panel b) a marked increase in site‐wide *Bromus* cover. The strongest 10% of links are drawn. We hypothesise that *Bromus* becoming widespread altered the competitive environment for *Plantago*, changing the geography of synchrony. These results were based on a subset of the data from Hallett et al. (2021).

More spatially complex changes in synchrony over time have less frequently been evaluated. Vindstad et al. (2019) considered temporal changes in the spatial directionality of synchrony (i.e., whether synchrony extends over longer distances in particular compass directions than others). They showed that temporal changes in the directionality of spatial synchrony in a moth species coincide with changes in the predominant direction of spring winds. A new analysis of synchrony in a long‐studied serpentine plant community (Hobbs and Mooney 1985; Walter et al. 2021) showed that the geography of synchrony of the historically competitively dominant native forb *Plantago erecta* changed markedly following invasion by the non‐native grass *Bromus hordeaceus* (Figure 5). Though Walter et al. (2017) provided detailed maps of large‐magnitude changes in the synchrony of a vegetation index across the entire continental United States (their figure 2), they did not explore possible reasons for the changes, which are likely multifarious. These examples illustrate how scrutinising long‐term data for changes in the geography of synchrony over time has yielded important insights into broad‐scale ecological processes, but that many research opportunities remain.

### 4.5 Benefits of Long‐Term Data for Detecting Changes in Synchrony Through Time

It is difficult to avoid the necessity of long‐term data for studying changes in synchrony through time for at least two reasons: (1) if some number of time steps are needed to characterise a pattern, a larger number is needed to characterise how that pattern has changed; and (2) longer time series are more likely to show changes, whether because changes occur slowly or because they are due to rare events. Though these reasons are logically straightforward, they are also fundamental and indicate the importance of long time series for facilitating major advances in ecology. Studies of directional changes of various kinds, due to climate change and other anthropogenic influences, are one of the central foci of modern ecology.

The changes discussed in this section could not have been detected using shorter time series that did not overlap the change and did not contain sufficient data before and after the event to conclude that dynamics had changed. Classic studies of changes in synchrony were based on extremely long time series, for instance, of rodents (Henttonen et al. 1987; Steen et al. 1990) or lynx and hare (Ranta, Kaitala and Lindstrom 1997). The example of changing geography of synchrony in a serpentine plant community (Figure 5) relied on a 37‐year time series, with the likely cause of the change—the marked increase in the prevalence of *Bromus*—occurring 17 years into sampling. Marked changes through time in synchrony were one of the main conclusions of the long‐term measles study by Grenfell et al. (2001) and an earlier, related study (Rohani et al. 1999) that used some of the same epidemiological data. Such changes are also visible for aphid phenology in figure 1 of Sheppard et al. (2016). Though ecologists would long have appreciated the likelihood that patterns of synchrony can change, many historical studies of synchrony used correlation‐based metrics of synchrony which tend to obscure such changes. Classic and modern studies based on long time series and using an ever‐expanding suite of statistical methods have greatly expanded conceptual frameworks around changes in synchrony through time. It is now recognised that not only are changes in synchrony important and common, they are also probably another of the major consequences of climate change (Hansen et al. 2020).

## 5 New Ecological Mechanisms Related to Synchrony

We review three recently discovered population‐dynamical mechanisms that relate to synchrony and then discuss how the discoveries of these mechanisms relied on long‐term data.

### 5.1 Interacting Moran Effects

Recent work demonstrated that Moran effects of distinct environmental variables acting on the same populations can interact, producing either more or less population synchrony than would be expected if the two drivers acted independently (Castorani et al. 2022; Reuman et al. 2023; Sheppard et al. 2019). Though it has long been recognised that multiple environmental drivers may shape synchrony (Kerlin et al. 2007; Moran 1953; Nicolau et al. 2022), only recently has a quantitative framework for investigating interactions between distinct Moran drivers been developed. (A different sort of interaction between Moran effects and dispersal was previously modelled theoretically by Kendall et al. 2000.) Following Reuman et al. (2023), we use a simple analogy to a playground swing‐set to convey the idea of how such interactions work. Imagine N children, each swinging on their own swing and representing, in this analogy, oscillating biological populations. Suppose each child is being pushed on their swing by both their own sister and their own brother, representing two distinct environmental influences. If the sisters (respectively, brothers) from separate families were to synchronise their pushes, it would produce a Moran effect (respectively, another separate Moran effect), tending to synchronise the swinging children. However, whether the synchrony produced by the sisters' Moran effect reinforces or counteracts that produced by the brothers' Moran effect depends on whether the sisters and brothers coordinate their pushes with each other. For instance, if the sisters and brothers are standing on the same side of the swinging children and their pushes are timed to coincide, then synchrony would be enhanced, in a synergistic Moran interaction. Antagonistic interactions are also possible (Castorani et al. 2022; Reuman et al. 2023).

**FIGURE 6:**
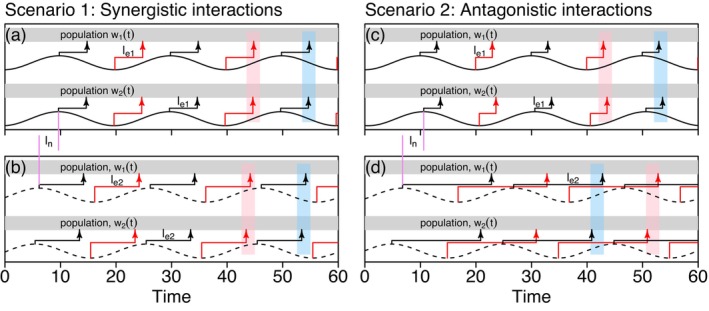
Figure illustrating the main idea of interacting Moran effects. If each of two environmental variables is itself spatially synchronous, then the degree of alignment of three lags determine the nature of interactions. Solid sine waves (a, c) represent the period‐20 components of an environmental driver in two locations ($\epsilon_{i}^{\left(1\right)}$ for i=1,2) and dashed sine waves (b, d) are the period‐20 components of a different driver in the same locations ($\epsilon_{i}^{\left(2\right)}$ for i=1,2). Black arrows are peak positive influences of environment on populations, lagged by $l_{e1}$ for $\epsilon_{i}^{\left(1\right)}$ and by $l_{e2}$ for $\epsilon_{i}^{\left(2\right)}$; these lags differ across the scenarios, but are the same across locations. Red arrows signify maximally negative effects. Peak positive effects of the same variable occur at similar times across locations, illustrated with rectangles, and corresponding to two Moran effects. In the synergistic scenario, the lag between the environmental variables ($l_{n}$) and the lags of their effects ($l_{e1}$ and $l_{e2}$) are aligned, so peak effects of $\epsilon_{i}^{\left(1\right)}$ coincide with peak effects of $\epsilon_{i}^{\left(2\right)}$, augmenting synchrony. In the antagonistic scenario, lags are misaligned. So peak positive effects of $\epsilon_{i}^{\left(1\right)}$ coincide with peak negative effects of $\epsilon_{i}^{\left(2\right)}$, and vice versa, reducing synchrony. Adapted with permission from Reuman et al. (2023).

The above analogy serves to render transparent the main idea of Moran interactions, but the greater challenge of applying this idea to real systems is facilitated by a quantitative, timescale‐specific theory (Reuman et al. 2023) and a suite of wavelet‐based statistical tools (Castorani et al. 2022; Reuman et al. 2021; Sheppard et al. 2019). Figure 6 moves this analogy closer to the case of populations. Both the swing‐set example and Figure 6 differ from real populations because they fluctuate on a single timescale, whereas real populations fluctuate on many timescales simultaneously. The recent theory and methods can be applied to decompose timescales, thereby considering separately each of the many pertinent timescales and again illustrating the importance of a timescale‐specific approach to synchrony.

### 5.2 Population Cycles and Synchrony

Classic theoretical and empirical studies have demonstrated a fundamental link between synchrony and population cycles (Bjørnstad 2000; Ranta et al. 1998). Populations that have cyclic dynamics can be more easily synchronised than those with chaotic or point attractors due to a phenomenon called ‘phase‐locking’ (Bjørnstad et al. 1999; Blasius et al. 1999; Blasius and Stone 2000). Phase‐locking of cyclic populations can stem from Moran effects, species interactions, and dispersal (Bjørnstad et al. 1999; Fox et al. 2011; Hopson and Fox 2019; Vasseur and Fox 2009; Wanner et al. 2024).

More recently, two long‐term studies have shown a kind of ‘converse’ to this classic result: populations that are synchronised only on certain timescales can thereby exhibit pronounced cyclic population dynamics on large spatial scales (Anderson et al. 2021; Emery et al. 2023). A key component of this realisation is that synchrony can occur preferentially at specific timescales (Anderson et al. 2021); timescale‐specific synchrony then leads to large spatial‐scale cyclic dynamics on the same timescales. Essentially, synchrony causes local fluctuations on the synchronised timescales to reinforce each other in the spatial total population time series, producing strong oscillations. We hasten to add that this newer result is by no means a formal, mathematical converse to the classic result; it is only superficially a ‘converse’ in the sense that cyclicity promotes synchrony in the classic result but synchrony promotes cyclicity in the new result, albeit under distinct circumstances.

**FIGURE 7:**
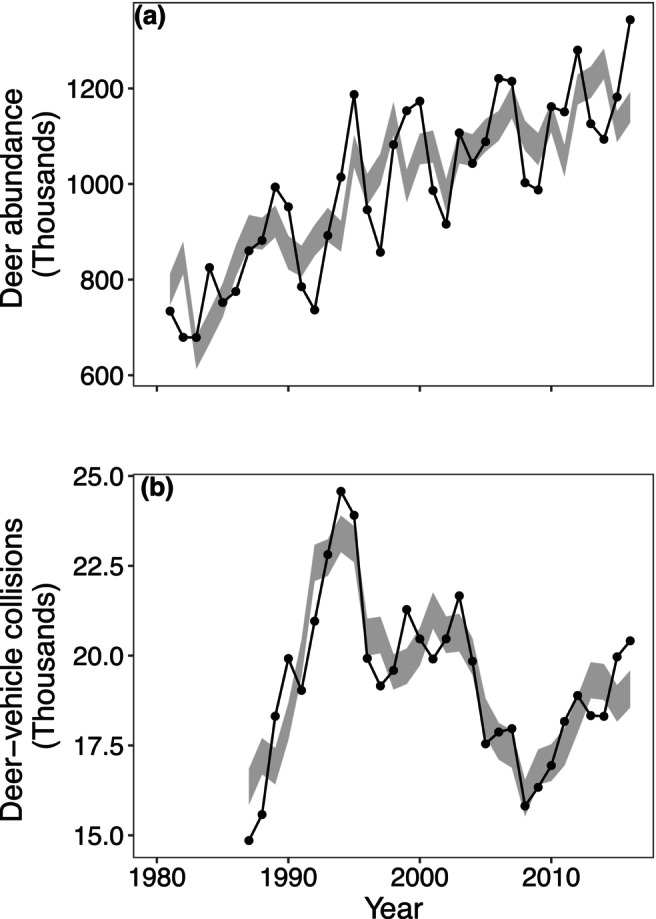
Fluctuations in deer abundance (a) and deer‐vehicle collisions (b) across a 36‐year period. The solid black lines indicate statewide totals across Wisconsin, USA; 3–7 year fluctuations are visible, though superimposed on a trend (a) or a longer‐timescale fluctuation (b). The grey band indicates the 95% quantiles of state‐total time series based on surrogate (i.e., appropriately randomised) county‐level time series modelling what would have occurred if 3–7 year synchrony between the county‐level time series were absent (Anderson et al. 2021) but these local fluctuations were otherwise statistically unchanged: 3–7 year fluctuations in state‐total time series are then absent or much reduced, simply because of the removal of 3‐ to 7‐year synchrony between the county‐level time series. This indicates how timescale‐specific synchrony helps produce the state‐level periodicity. This figure adapted from Anderson et al. (2021).

Careful examination of long time series of white‐tailed deer (*Odocoileus viriginianus*) populations in Wisconsin, USA, is what originally gave rise to the realisations described above (Anderson et al. 2021). Moran effects of winter weather and ENSO synchronised deer populations at timescales of 3–7 years (Figure 2e). Synchrony led to periodic dynamics in state‐total deer numbers that would not have been present had the populations not been synchronised (Figure 7a). Additionally, synchronised deer populations corresponded to synchrony in deer‐vehicle collisions over the same timescales (Figure 7b). Fluctuations were substantial, with swings of up to 250,000 deer and 2600 deer‐vehicle collisions between high and low years. Emery et al. (2023) demonstrated the same mechanism in cabbage‐stem flea beetles (*Psylliodes chrysocephala*).

### 5.3 Asymmetric Tail Associations

**FIGURE 8:**
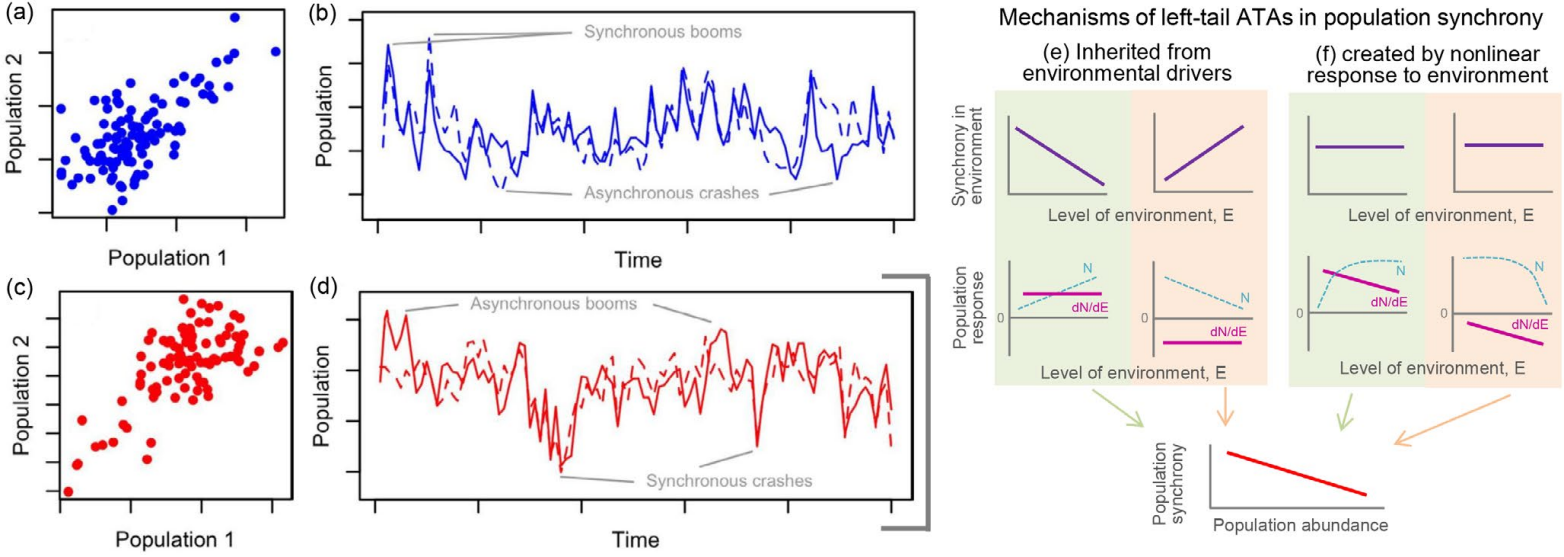
Asymmetric tail associations in population synchrony and their mechanisms. (a, b) Right‐tail ATAs lead to greater synchrony at higher population sizes, leading to synchronous population booms. (c, d) Left‐tail ATAs lead to greater synchrony at lower population sizes, leading to synchronous busts. Left‐tail ATAs with synchronous population crashes may arise from ATAs in underlying environmental drivers or from nonlinear population responses to the environment (e, f), see Section 5.3 for details. Panels (a–d) are reproduced from Walter et al. (2022).

Recent work highlights that the strength of population synchrony can depend on population abundance. Termed asymmetric tail associations (ATAs), right‐tail ATAs involve a greater level of synchrony at high population abundance (right tails) compared to low abundance. Right‐tail ATAs thus represent the case where populations have synchronous ‘booms’ and less synchronous ‘busts’ (Ghosh, Sheppard, Holder, et al. 2020; Figure 8a,b). Conversely, left‐tail ATAs involve a greater level of synchrony at low population abundance, representing synchronous busts and less synchronous booms (Figure 8c,d).

While both types of ATAs can be biologically important, left‐tail ATAs (spatially synchronous population busts) can increase the risk of metapopulation extinction (Ghosh, Sheppard, and Reuman 2020). Left‐tail ATAs in synchrony of resources (e.g., prey) or right‐tail ATAs in stressors (e.g., heat) can also disproportionately impact mobile consumers: when resources tend to be sparse everywhere, or conditions are stressful everywhere, movement is a weaker buffer against mortality.

Empirically, Ghosh, Sheppard, Reid, et al. (2020) found that a preponderance of either right‐ or left‐tail ATAs could occur among aphids of different species found in the same area; the same was true of marine algae (dinoflagellates) in UK seas. For giant kelp forests, right‐tail ATAs occurred in some sets of subpopulations while other sets exhibited left‐tail ATAs (Walter et al. 2022).

In general, ATAs in population synchrony can be (1) inherited from ATAs in the synchrony of environmental drivers and/or (2) created by nonlinear population responses to synchronous environmental drivers. Mechanism (1) happens, for instance, when populations respond approximately linearly to a synchronous environment with an ATA, and the environment is either (1.i) a population growth promoter that is more synchronised at low levels (i.e., a left‐tail ATA resource) or (1.ii) a growth inhibitor that is more synchronised at high levels (i.e., a right‐tail ATA stressor; Figure 8e; Ghosh, Sheppard, Holder, et al. 2020). The latter case may commonly arise in systems where stressors such as droughts or heatwaves span larger spatial scales when they are locally more extreme (Ghosh et al. 2021). Mechanism (2) happens, for instance, when a synchronous environment lacks ATAs and populations exhibit either (2.i) a saturating response to a growth promoter or (2.ii) an accelerating response to a growth inhibitor (Figure 8f; Ghosh, Sheppard, Holder, et al. 2020; Walter et al. 2022). In either case, populations are more sensitive to the environment—and thus inherit more synchrony from the environment—under adverse conditions. The kelp forest example introduced above (and explored in detail by Walter et al. 2022) provides an example of 2.ii, an ATA caused by a nonlinear response to a negative environmental force: among relatively sheltered locations, which have low mean wave height, years with low‐intensity storms have little negative effect on kelp such that population densities are generally high, but synchrony is limited because local factors influence dynamics; whereas years with high‐intensity storms reduce all populations simultaneously.

### 5.4 Benefits of Long‐Term Data for Understanding Mechanisms

For the themes of this synthesis, a key observation stemming from the case studies considered above is that careful examination of long time series can be a very effective means of awakening researchers to the importance of previously unnoticed mechanisms. The new mechanisms can then be theoretically studied and generalised. For all three cases above, the availability and careful statistical study of long time series was what enabled the discovery of the mechanisms described. This is distinct from an alternative pathway whereby a potential mechanism may first be described using models for subsequent testing with data.

For interacting Moran effects, Sheppard et al. (2019) first demonstrated synergistic Moran interactions of temperature and grazing on phytoplankton; those authors relied on decades‐long time series. Castorani et al. (2022) subsequently demonstrated both synergistic and antagonistic Moran interactions between the influence of nutrients and waves on giant kelp, again relying on decades‐long time series. Those empirical papers preceded a general theoretical understanding (Reuman et al. 2023). Many papers have investigated when and why Moran effects may be weaker or stronger than expected (e.g., Massie et al. 2015; Rogers and Munch 2019). Interacting Moran effects may be another important factor explaining such deviations.

Anderson et al. (2021) originally discovered the mechanism whereby timescale‐specific synchrony leads to large‐spatial‐scale population cycles through a detailed examination of long time series of county‐level deer abundances. They only later developed a theoretical understanding. Though the theoretical idea here is not likely to surprise, *post hoc*, experts in timescale‐specific approaches to time series analysis, the realisation that this particular mechanism applies to real populations can only spring from detailed analysis of long time series.

The importance of ATAs in ecology was also realised initially through examination of extensive data sets, subsequently followed by theoretical explorations. Ghosh, Sheppard, Holder, et al. (2020) examined multiple ecological data sets, exploring whether those data sets had asymmetric tail dependence of synchrony. Statistical detection of ATAs requires a fairly large data set, so the explorations of Ghosh, Sheppard, Holder, et al. (2020) relied on the availability of data. The empirical realisation that ATAs are common in ecology was then followed by theoretical explorations of their causes (Walter et al. 2022), and their importance for stability (Ghosh et al. 2021), extinction risk (Ghosh, Sheppard, and Reuman 2020) and other aspects of ecology (Albert and Reuman 2023). The key point, for the theme here, is that this process began with the long time series.

## 6 Discussion

There are both incremental and paradigm‐expanding benefits of long time series. Incremental benefits include (1) increased statistical power to detect subtler effects, (2) increased likelihood of observing rare events and (3) more precise estimates. Valuable conceptual advances also arise. We here described several paradigm expansions that occurred over the past ≈20 years associated with the emergence of new research areas and approaches to synchrony. Developments include: (1) Synchrony has important timescale structure; (2) inferences of the causes of synchrony are now relatively straightforward; (3) synchrony can change over time in strength, timescale structure or geography, and such changes can be driven by climate; (4) Moran drivers of synchrony can interact; (5) timescale‐specific synchrony can create population cycles at large spatial scales; and (6) synchrony can be stronger when populations are at high than low densities, or vice versa (i.e., ATAs). These realisations emerged through examination of long time series. It is difficult to imagine these shifts in understanding arising without sufficient long‐term data sets.

To improve science on synchrony, research institutions and funding agencies should support existing long‐term studies, initiate new ones and expand data accessibility. Many of the paradigm‐expanding long‐term studies on synchrony that we highlighted would not have been possible without sustained funding‐agency support, such as the U.S. Long Term Ecological Research program, the Landsat program, epidemiological records, the CPR survey and the Rothamsted Insect Survey. Without these and similar programs, it is challenging for investigators to continue or initiate multidecadal studies. Expanded support for long‐term data collection and dissemination will continue to conceptually transform our understanding of synchrony. Crucially, despite growing mandates from government funders and publishers to meet data‐accessibility standards, many long‐term data sets are not yet openly, freely and easily accessible, or are not available in a manner that makes them readily usable (e.g., poorly maintained, in proprietary formats, not interoperable, lacking standardised metadata, restricted for re‐use; Gries et al. 2018). As we have argued, open and accessible long‐term data sets do much more than support scientific transparency and reproducibility; they also facilitate new paradigm expansions (Reichman et al. 2011). As data sets grow longer, evaluation of funding renewal requests should increasingly scrutinise plans and procedures for making data accessible, commensurate with the increasing value of the data set.

Spatially replicated population studies of 2‐decade duration (the arbitrary cutoff used in this paper) remain uncommon (Clutton‐Brock and Sheldon 2010; Witman et al. 2015). For fast‐generation organisms, comparably useful time series can often be established in less time through faster sampling. For instance, novel findings arose about the transmission of synchrony across ecosystem boundaries using an 11‐year monthly time series describing the accumulation of detrital kelp wrack on beaches and the response of shorebirds to this resource subsidy (Walter et al. 2024). The rapid turnover of kelp and quick behavioural response of shorebirds to forage on wrack‐associated invertebrates allowed for the discovery of interacting, timescale‐specific drivers of synchrony with ‘only’ 11 years of data.

In the Introduction, we paraphrased Doak et al. (2008) suggesting that long‐term data sets are important for conceptual advances because ecological dynamics are driven by many mechanisms acting simultaneously, many of which may be unknown to science. We here extend those speculations. Suppose there are 10 major mechanisms or principles in a particular domain of knowledge, all of which are known to science, and that there are many systems for which only one or a few theories are important. An example of such a domain could be physics, where for major theories including Newtonian mechanics and relativity, systems abound which can be understood in detail through the application of that theory alone. On the other hand, imagine a different domain of knowledge for which there are 100 main principles and only 20 are known to science. Further suppose that any given system in this latter domain is simultaneously influenced by a substantial number of the 100 mechanisms. Ecology may be an example of such a domain. For these domains, trying to understand a new system by applying one of the 20 known theories may be futile if the dominant mechanisms for the system are not among those 20. Correspondingly, efforts to test existing major theories in ecology often conclude that the theory provides only partial insight, with boundaries unclear between when a theory can and cannot be expected to succeed in explaining data (Harrison 2017; McGill et al. 2006; Price et al. 2012; Ricklefs 2006). While efforts to pose and test theories will remain important in ecology, and theories can provide insight when they fail as well as when they succeed, long‐term data provide an alternative approach. As we have shown here for synchrony, analysis of long‐term data can reveal previously unknown mechanisms which may be among those confounding the application of existing theories.

## Author Contributions

D.C.R., J.A.W. and M.C.N.C. led the paper. All authors contributed text or figures to individual sections, and all authors contributed to editing.

## Supporting information


Data S1.

## Data Availability

All codes and necessary data associated with this study have been released on Zenodo, https://doi.org/10.5281/zenodo.14976903, as well as in a public GitHub repository, https://github.com/reumandc/SpatialSyncAndLongTermData.
