# Supplementary material for: Insights Into Spatial Synchrony Enabled by Long‐Term Data
Source: Ecol Lett. 2025 Apr 23;28(4):e70112. doi: 10.1111/ele.70112 (PMC12018873; doi:10.1111/ele.70112)
Supplement: Supplementary file 1 — Data S1. [file ELE-28-0-s001.pdf]

Supporting information for: Insights into spatial synchrony enabled by long-term data

Daniel C. Reuman[1], Jonathan A. Walter[2,3], Lawrence W. Sheppard[4], Vadim A. Karatayev[1], Ethan S. Kadiyala[3], Amanda C. Lohmann[3], Thomas L. Anderson[5], Nat J. Coombs[1], Kyle J. Haynes[3], Lauren M. Hallett[6], and Max C.N. Castorani[3]

[1]Department of Ecology & Evolutionary Biology and Center for Ecological Research, University of Kansas
[2]Center for Watershed Sciences, University of California, Davis
[3]Department of Environmental Sciences, University of Virginia
[4]Marine Biological Association of the United Kingdom
[5]Department of Biological Sciences, Southern Illinois University, Edwardsville
[6]Department of Biology, University of Oregon

1 Methods details for Fig. 2

Bristlecone pine growth time series on which Fig. 2h was based were obtained as follows. Raw bristlecone pine (*Pinus longaeva*) tree ring measurements were acquired from the International Tree-Ring Data Bank. Data were queried by filtering "Tree Species" to "PILO," the code for the species, yielding 40 studies. The following nine studies, corresponding to distinct stands, were chosen based on temporal extent: ca534, ca535, ca667, nv515, nv516, nv520, nv521, ut509, and ut550 (Bekker *et al.*, 2021; Graybill, 2002a,b,c,d,e; Salzer & Hughes, 2010a,b,c). The raw ring width measurements (.rwl files) were downloaded and subsequently processed in R v4.1.0 using the *dplR* package (Bunn *et al.*, 2023; R Core Team, 2021). Following dendrochronological best practices (Bunn, 2008), we constructed growth chronologies for the nine corresponding stands of bristlecone pine trees, consisting of 508 samples spanning 1980 years and 550 km of spatial extent across California, Nevada, and Utah.

First, raw tree ring measurements were parsed into R, converting Tucson formatted .rwl files to data frames. For each sample in a site, raw tree ring width measurements were then detrended to adjust for biological and stand effects by fitting a negative exponential curve of the form $y_t = a\exp(-bt) + k$ to the series, where y_t is the expected growth at year t (Frites,

2001) and the values of a, b, and k are parameterized to each series. We then calculated a dimensionless ring-width index by dividing each series by the growth trend estimated by the fitted curves. We then produced mean-value chronologies by averaging ring-width indices across series within a site using Tukey's biweight robust mean (Frank *et al.*, 2022).

Growth chronologies for all nine sites were then trimmed to the same temporal extents, beginning in year 0 CE and ending in 1979 CE. The sample depth (number of trees) for each growth estimate was lower for earlier years, however nearly all of our 17820 observations (99.9%) had more than one sample, and the vast majority (91.0%) had at least 10 samples.

The resulting nine time series of bristlecone pine mean growth chronologies were then further processed and analyzed using the *wsyn* package Reuman *et al.* (2021). Data were cleaned by applying an optimal Box-Cox normalization procedure to each time series. Transformed time series were also individually linearly detrended, de-meaned, and variances were standardized to 1. These steps were carried out using the cleandat function in the *wsyn* package. Wavelet phasor mean fields were then calculated using the *wpmf* function in *wysn*. The output was plotted to depict synchrony over time and timescale, with contours indicating statistically significant values of synchrony at the 95% level.

Car accident mortality time series on which Fig. 2b was based were obtained as follows. Raw monthly car accident mortality time series by state were taken from the Multiple Cause of Death database via CDC Wonder. Data were queried by setting "Group Results by" to "State", "Year", and "Month" and filtering "Injury Intent and Mechanism" by "Unintentional" and "Motor Vehicle Traffic". The resulting time series were then downloaded and processed in R v4.1.0 (R Core Team 2021). Due to privacy law, additional processing was required to address suppressed values (where < 10 deaths would otherwise have been reported). Time series with $> 25\%$ suppression were removed, and remaining suppressed values were replaced with 5, a central value in the suppressed range $0 - 10$.

The resulting time series were then processed and analyzed similarly to the bristlecone growth chronologies using the *wsyn* package (Reuman *et al.*, 2021). An optimal Box-Cox normalization procedure was applied to each time series individually, followed by individually detrending, demeaning, and standardizing the variances to 1. Then, wavelet phasor mean fields were calculated and plotted exactly as described above using *wsyn*.

2 Reasons for small differences between Fig. 2 and previously published results

Fig. 2a matches approximately, but not exactly, with Fig. 2a of Walter *et al.* (2024) because the figure of this study used the wavelet phasor mean field, and the figure of Walter *et al.* (2024) used the wavelet mean field, a different but related technique.

Fig. 2c matches approximately, but not exactly, with Fig. 3a of Castorani *et al.* (2022) because this study used the wavelet phasor mean field, and the earlier study used the wavelet mean field.

Fig. 2d does not match well with Fig. 1a of Sheppard *et al.* (2016) because Fig. 2d

describes the synchrony of the willow-carrot aphid, and Fig. 1a of Sheppard *et al.* (2016) describes the average synchrony of 20 aphid species. Also, Fig. 2d of this study used the wavelet phasor mean field, and Fig. 1a of Sheppard *et al.* (2016) used the wavelet mean field.

Fig. 2e matches well, but not perfectly, with Fig. 3b of Anderson *et al.* (2021). Colors are the same, but contours differ slightly because a 95% significance threshold was used here and a 99.9% threshold was used by Anderson *et al.* (2021).

Fig. 2f matches Fig. 2a of Sheppard *et al.* (2019) approximately, but not exactly, because the figure of this study used a wavelet phasor mean field, and figure of Sheppard *et al.* (2019) used a wavelet mean field.

Fig. 2g looks similar, but not identical to Fig. 2b of García-Carreras *et al.* (2022) because the figure of this study used a wavelet phasor mean field and García-Carreras *et al.* (2022) used a wavelet mean field; and the colormaps of the two figures also differed.

3 Statistical significance of timescale structure in synchrony

For consistency and to clearly present main ideas, we focused in the main text on a single method of displaying time and timescale structure in synchrony, the wavelet phasor mean field (wpmf). Though the method provides quantification of one kind of statistical significance, additional analyses and arguments, exploiting additional data, have typically also been used to robustly support the conclusion that timescale structure in synchrony is important in a given system. Though these additional analyses have varied by system, we here summarize the analyses for a few of the studies discussed in section 2.2 of the main text and for which wpmfs are pictured in Fig. 2, starting with the study of Sheppard *et al.* (2016). It is important to note that wavelet mean field plots as shown in Fig. 2 do not, on their own, demonstrate that the differences in strength of synchrony between timescales are statistically significant. However, the timescale-specific features found in the synchrony of these systems have generally been found to be statistically associated with particular drivers of synchrony at the associated timescales (in the corresponding references, Fig. 2 caption). The nature of some of these detailed investigations is what is discussed below.

Sheppard *et al.* (2016) had access to population time series for 20 aphid species, and also to environmental data for the locations and times of the population measurements. Paired t-tests across species revealed that average values of mean field plots, across times before 1993 and "long" timescales ($>$ 4 years), were significantly greater than average values across times before 1993 and "short" timescales ($<$ 4 years). This reveals significant timescale structure in synchrony, prior to 1993, because if mean field patterns were solely the result of sampling variation, consistencies across species such as those demonstrated by this test would have been highly unlikely. The inference, here, is not based solely on interpretations of any single mean field plot, but draws inferential strength from the availability of multi-species data. Analogous paired t-tests across species showed that long-timescale synchrony dropped

significantly and short-timescale synchrony increased significantly from before 1993 to after, with the result that short-timescale synchrony was significantly stronger than long-timescale synchrony after 1993. So consistencies across species showed not only that timescale structure in synchrony was present, but also that it changed at about 1993.

Sheppard *et al.* (2016) also exploited available environmental data to strengthen inferences. Those authors used formal tests based on wavelet coherence to provide strong evidence that synchrony in winter temperature across their sampling locations was the cause of the large majority of observed aphid synchrony. The spatial coherence metrics of Sheppard *et al.* (2016) measure to what extent a population variable and an environmental variable have consistent phase differences and correlated magnitudes of oscillation over time at all sampling locations, as a function of timescale. As oscillations in a population variable typically cannot plausibly cause environmental oscillations, a significant spatial coherence on a given timescale band shows a probable causal effect of the environmental variable, or another one closely related with it, on the population variable on that band. This is because irregular oscillators that are not actually related are unlikely to maintain consistent phase differences or magnitude correlations. Timescale variation and changes through time in synchrony were thus significantly related to time-timescale patterns of synchrony in winter temperature. If population mean field patterns were solely the result of sampling variation, such consistencies with winter temperature would have been highly unlikely; and so mean field patterns were unlikely to be solely due to sampling variation.

Thus, the overall conclusion based on Sheppard *et al.* (2016) that timescale structure in population synchrony is meaningful and important for their system is illustrated and partly supported by patterns in the wpmf plot, but is provided with substantial additional formal statistical support through a variety of carefully considered tests making use of additional data beyond the population time series themselves.

Several other published studies have also used coherence-based tests to trace time/timescale features in population synchrony back to environmental drivers, thereby showing the high unlikelihood that the features arise solely from sampling variation. The timescale band of high synchrony in deer populations in main text Fig. 2e was found to be related to climate variability with the same timescale. And the timescale of the large, long-timescale feature in synchrony of Plankton Colour Index variability (main text Fig. 2e) was explained by timescale-specific variability of temperature and zooplankton variables. Castorani *et al.* (2022) and Walter *et al.* (2024) took a similar approach as well.

Published analyses such as those sketched above, based on additional data beyond just population time series, are part of the argument for timescale structure in all of the examples illustrated in Fig. 2, except for the car crash and bristlecone pine examples (panels b and h) which have not been published elsewhere. It could be the case, *a priori*, that the synchrony depicted in those panels is not significantly structured by timescale if an appropriate null hypothesis can be determined for formally testing that with only population data. It is also possible that analyses analogous to those sketched above would reveal that the timescale structure seemingly illustrated in those panels is significant and meaningful.

References

Anderson, T. L., Sheppard, L. W., Walter, J. A., Rolley, R. E. & Reuman, D. C. (2021). Synchronous effects produce cycles in deer populations and deer-vehicle collisions. *Ecology Letters*, 24, 337–347.

Bekker, M., DeRose, R., Gray, H., Wortham, S. & Jess, R.and Wheeler, S. (2021). NOAA/WDS Paleoclimatology - Bekker - Red Canyon - PILO - ITRDB UT550. URL https://doi.org/10.25921/6h43-0334.

Bunn, A. (2008). A dendrochronology program library in r (dplr). *Dendrochronologia*, 26, 115–124.

Bunn, A., Korpela, M., Biondi, F., Campelo, F., Mérian, P. & Qeadan, F. (2023). dplr: Dendrochronology program library in r. URL https://cran.r-project.org/web/packages/dplR/index.html.

Castorani, M. C. N., Bell, T. W., Walter, J. A., Reuman, D. C., Cavanaugh, K. C. & Sheppard, L. W. (2022). Disturbance and nutrients synchronise kelp forests across scales through interacting moran effects. *Ecology Letters*, 25, 1854–1868.

Frank, D., Fang, K. & Fonti, P. (2022). Dendrochronology: Fundamentals and innovations. In: *Stable Isotopes in Tree Rings: Inferring Physiological, Climatic and Environmental Responses*, Tree Physiology. Springer International Publishing, pp. 21–59.

Frites, H. (2001). *Tree rings and climate*, vol. 8. Blackburn Press.

García-Carreras, B., Yang, B., Grabowski, M. K., Sheppard, L. W., Huang, A. T., Salje, H., Clapham, H. E., Iamsirithaworn, S., Doung-Ngern, P., Lessler, J. *et al.* (2022). Periodic synchronisation of dengue epidemics in thailand over the last 5 decades driven by temperature and immunity. *PLoS biology*, 20, e3001160.

Graybill, D. (2002a). NOAA/WDS Paleoclimatology - Graybill - Hill 10842 Recollection - PILO - ITRDB NV516. URL https://doi.org/10.25921/2ak6-tq38.

Graybill, D. (2002b). NOAA/WDS Paleoclimatology - Graybill - Indian Garden - PILO - ITRDB NV515. NOAA National Centers for Environmental Information. URL https://doi.org/10.25921/r81r-sw85.

Graybill, D. (2002c). NOAA/WDS Paleoclimatology - Graybill - Mammoth Creek - PILO - ITRDB UT509. URL https://doi.org/10.25921/9npw-ma05.

Graybill, D. (2002d). NOAA/WDS Paleoclimatology - Graybill - Methuselah Walk - PILO - ITRDB CA535. URL https://doi.org/10.25921/ppqj-xv48.

Graybill, D. (2002e). NOAA/WDS Paleoclimatology - Graybill - Sheep Mountain California - PILO - ITRDB CA534. URL https://doi.org/10.25921/q0we-8b37.

R Core Team (2021). R: A language and environment for statistical computing. URL https://www.R-project.org/.

Reuman, D., Anderson, T., Walter, J., Zhao, L. & Sheppard, L. (2021). wsyn: Wavelet approaches to studies of synchrony in ecology and other fields. URL https://CRAN.R-project.org/package=wsyn.

Salzer, M. & Hughes, M. (2010a). NOAA/WDS Paleoclimatology - Salzer - Mount Washington Nevada Update - PILO - ITRDB NV520. URL https://doi.org/10.25921/21ew-pa87.

Salzer, M. & Hughes, M. (2010b). NOAA/WDS Paleoclimatology - Salzer - Pearl Peak Update - PILO - ITRDB NV521. URL https://doi.org/10.25921/rf2e-kb29.

Salzer, M. & Hughes, M. (2010c). NOAA/WDS Paleoclimatology - Salzer - Sheep Mountain Update - PILO - ITRDB CA667. URL https://doi.org/10.25921/rf2e-kb29.

Sheppard, L. W., Bell, J. R., Harrington, R. & Reuman, D. C. (2016). Changes in large-scale climate alter spatial synchrony of aphid pests. *Nature Climate Change*, 6, 610–613.

Sheppard, L. W., Defriez, E. J., Reid, P. C. & Reuman, D. C. (2019). Synchrony is more than its top-down and climatic parts: interacting moran effects on phytoplankton in british seas. *PLoS Computational Biology*, 15, e1006744.

Walter, J. A., Emery, K. A., Dugan, J. E., Hubbard, D. M., Bell, T. W., Sheppard, L. W., Karatayev, V. A., Cavanaugh, K. C., Reuman, D. C. & Castorani, M. C. N. (2024). Spatial synchrony cascades across ecosystem boundaries and up food webs via resource subsidies. *Proceedings of the National Academy of Sciences*, 121, e2310052120.
